# Plant-Based Meat Analogues in the Human Diet: What Are the Hazards?

**DOI:** 10.3390/foods13101541

**Published:** 2024-05-15

**Authors:** Maria Gräfenhahn, Michael Beyrer

**Affiliations:** Institute of Life Technologies, University of Applied Sciences and Arts Western Switzerland Valais-Wallis (HES-SO VS), 1950 Sion, Switzerland

**Keywords:** plant-based meat analogues, nutritional hazards, microbial aspects, extrusion processing, chemical compounds, processing compounds

## Abstract

Research regarding meat analogues is mostly based on formulation and process development. Information concerning their safety, shelf life, and long-term nutritional and health effects is limited. This article reviews the existing literature and analyzes potential hazards introduced or modified throughout the processing chain of plant-based meat analogues via extrusion processing, encompassing nutritional, microbiological, chemical, and allergen aspects. It was found that the nutritional value of plant-based raw materials and proteins extracted thereof increases along the processing chain. However, the nutritional value of plant-based meat analogues is lower than that of e.g., animal-based products. Consequently, higher quantities of these products might be needed to achieve a nutritional profile similar to e.g., meat. This could lead to an increased ingestion of undigestible proteins and dietary fiber. Although dietary fibers are known to have many positive health benefits, they present a hazard since their consumption at high concentrations might lead to gastrointestinal reactions. Even though there is plenty of ongoing research on this topic, it is still not clear how the sole absorption of metabolites derived from plant-based products compared with animal-based products ultimately affects human health. Allergens were identified as a hazard since plant-based proteins can induce an allergic reaction, are known to have cross-reactivities with other allergens and cannot be eliminated during the processing of meat analogues. Microbiological hazards, especially the occurrence of spore- and non-spore-forming bacteria, do not represent a particular case if requirements and regulations are met. Lastly, it was concluded that there are still many unknown variables and open questions regarding potential hazards possibly present in meat analogues, including processing-related compounds such as n-nitrosamines, acrylamide, and heterocyclic aromatic amino acids.

## 1. Introduction

To reduce possible health effects associated with meat, it has been argued in several developed countries that its consumption should be lowered [1]. Additionally, to match the nutritional needs of an expected increased world population, it is estimated that the production of meat will have to be doubled. However, the lack of resources, increased awareness of animal welfare, environmental and climate changes, and overall health, might led to a change in consumer behavior toward a reduction in the consumption of meat [2]. Consequently, alternative protein sources and processes are being implemented to manufacture alternatives to replace meat and related products. Meat analogues including cultured meat and plant-, insect-, and single-cell-based products can be manufactured to imitate the taste and texture of burgers, whole-cut meats, and sausages, among others. Alternative protein sources, including microalgae, seaweed, fungi, and insects are gaining interest due to their high nutritional value, high protein content, and potential sustainability and affordability. Even though their usage in food products is, in some cases limited, extracting their proteins increases their field of application and offers the possibility to produce ingredients with improved techno-functionality [3,4,5,6,7]. To achieve the desired sensory characteristics, meat analogues are mostly produced using proteins extracted from these sources [8,9].

**Figure 1:**
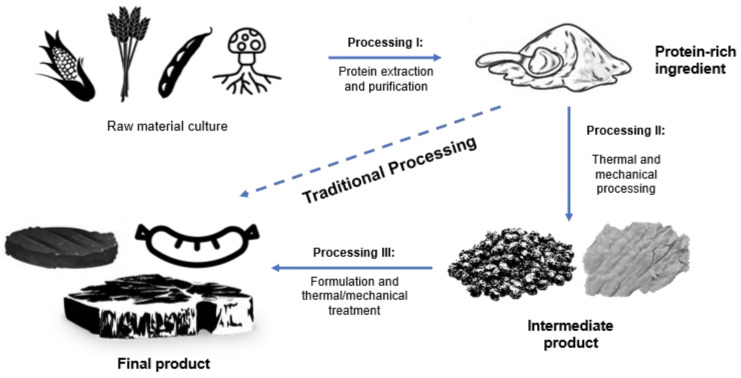
Schematic representation of the processing chain of meat analogues.

In Figure 1, a schematic representation of a possible processing chain for the production of meat analogues is depicted. There are different steps including the culture of the raw material, protein extraction, thermal and mechanical processing, and (re)-formulation. Since the cultivation step is a complex topic on its own it will not be considered in this review. The starting point are consequently the raw materials obtained after harvesting, e.g., peas and soybeans. The processing conditions during the protein extraction from these sources depend on the raw material itself and the desired protein quality. 

Protein extraction can be based on dry or wet-extraction methods and include milling, sieving, hydration steps, variation of pH conditions, or thermal treatment. These steps can modify the physical–chemical properties of the extracted proteins and, accordingly, their techno-functionality. Another method that, has gained interest in recent decades is fermentation combined with heat treatment, which is used to produce, among others, mycoproteins. In general, ingredients with high protein purity that are free from natural anti-nutritional or auxiliary (chemical) compounds and pathogens are desired. Therefore, information regarding which processing steps are needed for, e.g., the inactivation or partial removal of anti-nutritional factors is needed. Moreover, little information is available on the effect of processing on other contaminants such as pesticides or heavy metals [10]. Protein powders typically contain non-protein compounds, and the exact nature and concentration depend on the protein source and extraction method and conditions. Thus, a plant protein isolate contains approx. 10–20% non-protein compounds, including carbohydrates and fats, but also traces of contaminants, plant-originated anti-nutritional factors, or substances with toxic potential, when ingested at elevated concentrations. These non-protein compounds are not only present in products from alternative sources, but can also be present in, e.g., whey protein isolates. After the extraction, additional processing and formulation steps might be executed depending on the properties of the protein and the final product’s specifications. Traditional meat processing to manufacture e.g., sausages, results in the modification of meat properties by mincing, grinding, chopping, salting, and curing, the addition of seasonings and other food ingredients, and, in many instances heat treatment [11]. Therefore, the modification of the meat’s properties is often a consequence of the thermal and mechanical treatments, to which meat products are subjected. Consequently, orientated to this process, manufacturing steps to obtain meat analogues include thermal and/or mechanical processing of protein-based formulations e.g., extrusion. Extrusion processing is used to manufacture plant-based meat analogues. During this process, protein-rich raw materials are mixed with water, heated, sheared in the extruder barrel, and finally pushed through a die. The processing conditions lead to a change in the overall structure of protein-based ingredients and, therefore, in their properties. Plant-protein-based formulations are modified to resemble the structure and properties of various meat products. Most of the products obtained via extrusion processing are, however, intermediate products lacking the desired final texture. Therefore, they might be mixed with additional extracted proteins and be further processed using known equipment and conditions related to the meat industry to obtain e.g., plant-based nuggets, burgers, or sausages. However, these products often need many additional ingredients and additives for flavoring, coloring, and binding [3]. Vitamins and minerals are added to the formulation to make up for deficiencies in these alternative protein matrices as compared with meat. Additionally, antioxidants, organic acids, and phosphate compounds are added to reduce oxidation and rancidity and improve microbial stability and overall shelf life. In an overly simplistic manner, it can be understood that thermomechanical treatment is needed for cooking and texture development, whereas flavor, taste, and color, are modified with further ingredients in the formulation of the final food product.

Overall, the raw materials used to produce meat analogues are usually approved as “GRAS”, i.e., generally recognized as safe [9]. However, they could have e.g., nutritional drawbacks, which should be discussed and reviewed critically. Research regarding these products has been focused on formulation and process development to enhance food’s texture and overall sensory. Since meat analogues have been on the market for a relatively short time, research concerning their safety, shelf life, and long-term nutritional and health effects is limited. Thus, there might be overlooked aspects affecting human health. Therefore, the investigation of hazards related to the manufacturing of plant-based meat analogues and their consumption is the focus of this study. Additionally, this study is designed to examine changes in hazards or introduction of new possible hazards along the processing chain. It is expected that some hazards remain similar to those of meat products, but some new or still unknown hazards may arise specifically for meat analogues.

## 2. Plant-Based Protein Sources

Most plant-based protein sources, like soy and quinoa, have been widely consumed around the globe for many centuries. Furthermore, grains (e.g., wheat) and legumes (e.g., beans, lentils, peas, and lupins) are sources of proteins that can be sustainably produced. Although meat analogues can be produced using raw materials, i.e., peas or lentils, an increasing number of commercially available products include protein-based ingredients that have been refined to substitute animal proteins in the human diet. In various categories of meat analogues including burgers and sausages, the protein content ranges from 3–36 g protein per 100 g product [11,12].

The protein source in plants is mostly present in the seeds. Depending on the plant, the protein-rich sources are, therefore, beans, grains, or nuts, and they are usually covered with cellular structures like hulls, husks, or shells. To ensure long stability, the protein bodies can have a compact form consisting of storage proteins, i.e., prolamins, glutelins, and globulins. Albumins, also storage proteins, include protease and amylase inhibitors and lectins, which are undesirable due to their anti-nutritional properties [13,14]. To obtain protein-based ingredients, a multistep extraction process is often implemented. The extracting conditions depend on the raw material composition, i.e., protein, oil, and starch content, which also influences the nutritional potential of plant-based proteins [10,14]. 

**Figure 2:**
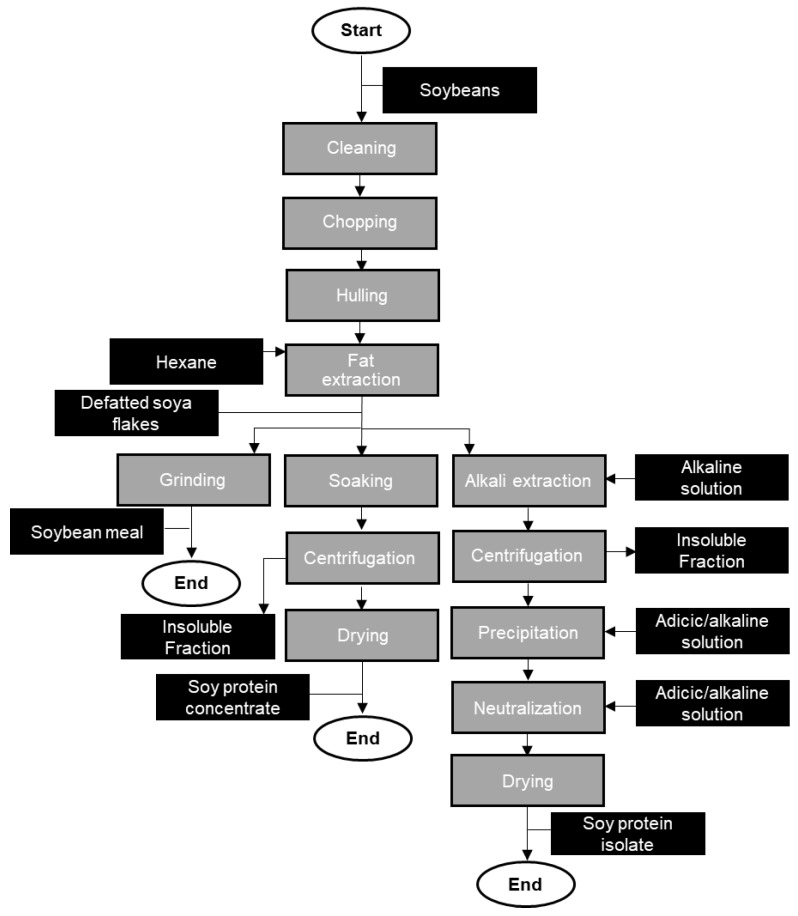
Processing flow to manufacture soy proteins.

**Figure 3:**
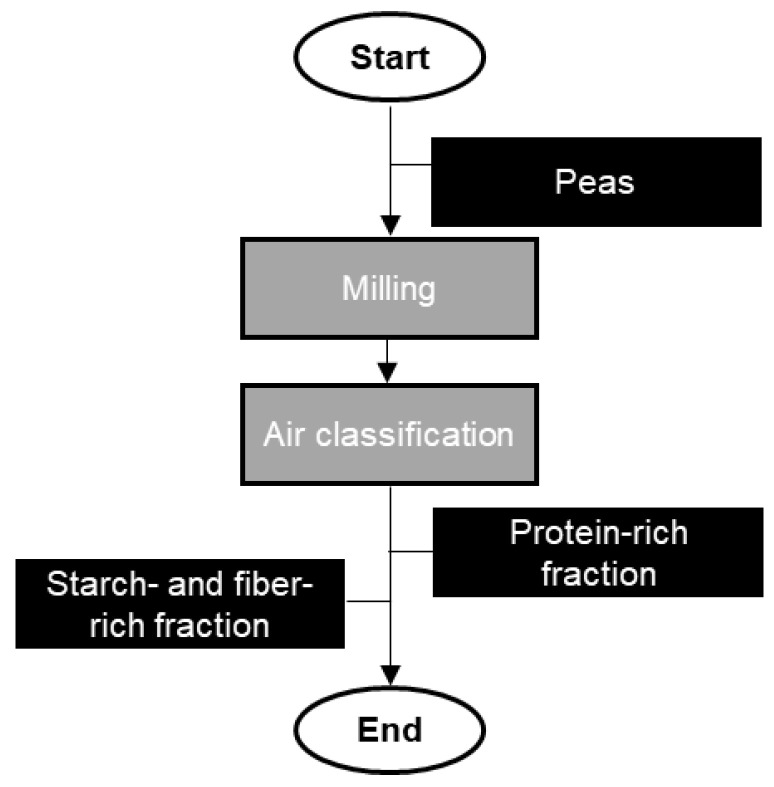
Processing flow to manufacture pea proteins.

The protein extracted from plants is produced using either wet fractionation (Figure 2) or dry fractionation (Figure 3) [10].

Wet fractionation is chosen for the manufacturing of protein isolates, mainly from legumes and oilseeds, e.g., soybeans or canola. In Figure 2, an exemplary process flow to produce soybean meal, soy protein concentrate, and soy protein isolate is depicted. For protein extraction from oil seeds, usually, a defatting step is added after cleaning, chopping, and hulling. Solvents like petroleum ether, n-hexane, and n-pentane are used during the defatting step to remove most of the oil present since it might interfere with protein extraction [15]. If no defatting step is necessary, the extraction process includes grinding, a hydration step with a pH shift, and a decanting or centrifugation step to remove the starch and insoluble fiber. Thereafter, protein precipitation, separation, and concentration are performed. The protein is precipitated, either by chemicals and solvents or by isoelectric point precipitation and recovered by centrifugation [15]. Isoelectric precipitation is often used for legumes to extract the globulin protein fractions, followed by a spray-drying step [10]. The manufacturing of pea protein isolate follows a similar approach, without the defatting step. 

Dry extraction or -fractionation is an alternative extracting method in which sieving and/or air classification techniques are combined to prepare fiber- and protein-rich fractions (Figure 3). For grains and cereals, the very first step in protein extraction is milling the plant source to release the protein bodies from the cells. Since the released material is a mixture of proteins with other macromolecules, including polysaccharides and lipids, the next steps are focused on the separation of the targeted proteins from the mixture of molecules [16]. This technique is mainly applied for starch-containing crops such as pea or fava beans, to obtain protein concentrates from flour.

Protein content depends on the protein and starch content of the beans or seeds, the differences in size between the protein body and the starch granules, and the milling efficiency [10]. After the extraction of the plant-based protein ingredients, they are transformed into meat-like structures via extrusion processing and then depending on the final product they imitate, the extruded proteins are mixed with the rest of the ingredients for the final formulation. 

## 3. Extrusion Processing of Plant-Based Meat Analogues

Extrusion is commonly defined as the operation of forming and shaping a molten or dough-like material by forcing it through a restriction or die [17]. During extrusion processing, proteins are exposed to heat and shear, which can lead to changes in the protein structure. Protein powder is fed into the barrel of the extruder, where it is mixed with water to form a proteinaceous dough. Heat transfer from the barrel, conversion of mechanical energy into thermal energy, and shearing from the rotation of the screws result in thermal and mechanical stresses acting simultaneously on the protein matrix during processing. Processing conditions for protein extrusion usually involve high temperatures and short times (up to two minutes). Depending on the desired texture to be achieved, either low or high moisture extrusion is implemented [18]. During low moisture extrusion (LME), processing conditions include temperatures between 80 and 180 °C and moisture contents from 10 to 40%. Higher moisture contents, i.e., 40 and 70%, and typically temperatures between 120 and 160 °C are chosen during high moisture extrusion (HME). The combination of heat and shear during extrusion processing causes the de-aggregation of globular proteins, unfolding, rearrangement, and aggregation into a cross-linked structure [19,20,21,22]. The most common and most thoroughly investigated extrusion process is carried out at low moisture levels (<40%) and is widely used in the food industry to produce texturized vegetable protein (TVP) [23]. TVP is produced mostly from defatted soy meal, soy protein concentrate, or wheat gluten [24]. These products expand in and after leaving the die due to water evaporation and therefore must be rehydrated for consumption [18]. After rehydration, the texture changes from brittle and hard to elastic and spongy. Consequently, they can be used in e.g., stews and sauces as a replacement for minced meat. In contrast, the extrusion process operated at moisture contents of 40% and higher is often used to produce meat analogues that resemble meat [25,26,27,28]. To avoid the expansion of the protein matrix, it is cooled down to below 100 °C before exiting the die. During the cooling down of the protein matrix in the die, a fibrous or layered structure develops which intends to mimic the meat structure. Even though the actual mechanisms leading to this fibrous structure are not fully understood, there are various theories previously reviewed in detail [29,30]. 

Plant-based products resembling minced meat products aim to recreate their texture, chewiness, and juiciness. Consequently, the extruded proteins are mixed with the rest of the ingredients to achieve the final product, which could be a plant-based burger or nugget. Commercially available plant-based burgers or nuggets include water, proteins, fats, carbohydrates (e.g., modified and/or resistant starches, dietary fibers, methylcellulose, carrageenan, calcium alginate, pectin, or konjac), salts (e.g., sodium, phosphate, and nitrite/nitrate salts), spices, and colorants and flavors (e.g., leghemoglobin protein). The different carbohydrates are added due to their water- and oil-holding capacity and gelling and thickening properties, which might lead to improved textural properties. As an alternative, the plant-based protein source can be added as a concentrate or isolate to enhance the functionality of the protein formulation, e.g., wheat gluten is added to plant-based burger formulations for its binding properties [31]. In contrast, to imitate whole-cut meats, like chicken or beef steak, meat analogues produced by HME are often chosen. They are then further processed by freezing, curing, marinating, and cooking to improve, among other aspects, the final structure, flavor, and color of the product [31]. Therefore, compared to plant-based burgers and nuggets, the formulation of whole-cut pieces, does not include as many ingredients to mimic the properties of the animal-based product.

## 4. Potential Hazards in Plant-Based Meat Analogues

Meat is known to have high protein digestibility and bioavailability; however, proteins extracted from, e.g., legumes, do not, and this might be a hazard. Several biological contaminants are related to plant-based proteins. Biological contaminants include bacteria, viruses, and parasites. The contaminants present in products which are consumed by humans should be controlled, since they may lead to foodborne illness outbreaks and even deaths. Millions of infections annually originate from foodborne pathogens such as *Campylobacter* spp., *Salmonella* spp., and *Escherichia coli*. These pathogens can be transferred to humans via meat products. In contrast, the human-animal interaction and by this the risk of zoonoses and infectious diseases decreases during the production of plant-based and singe cell proteins [8]. However, depending on the raw material used, i.e., biomass or protein powders, the risk may increase. Moreover, the hazards are transmitted from the raw material to the end products, and they are influenced by the many processing steps implemented to produce meat analogues. Considering the different thermomechanical treatments involved in the processing chain, it is expected that processing affects these protein-rich ingredients in a very similar manner to the influence of heat treatment on meat products. Some processing-related chemical contaminants that have been in the spotlight due to their presence in meat products in recent years, i.e., heterocyclic aromatic amines, N-nitrosamines, and polycyclic aromatic hydrocarbons, have not been mentioned frequently for meat analogues, even though the presence of these compounds in processed meat, has led to them to be labeled as carcinogenic to humans. Since plant-based protein sources also contain nitrogen compounds, amines, and reducing sugars, and the conditions used during extrusion processing include temperatures above 150 °C, it is possible that these toxic chemical compounds are also formed in intermediate meat analogues [1,32,33,34,35,36,37,38]. Furthermore, additional ingredients in the final formulation also carry their own risks, which also should be evaluated. 

## 5. Hazard Assessment

Eighteen different hazards related to soy- and pea-based meat analogues were considered in this review and they belong to four categories: chemical, microbiological, and nutritional hazards. Allergens were considered as an additional category. A scoring system ranking the found hazards in levels from low to high was implemented from the found literature. The scoring system results when both parameters, i.e., probability of occurrence (Table 1) and impact on human health (Table 2), were considered. 

**Figure 4:**
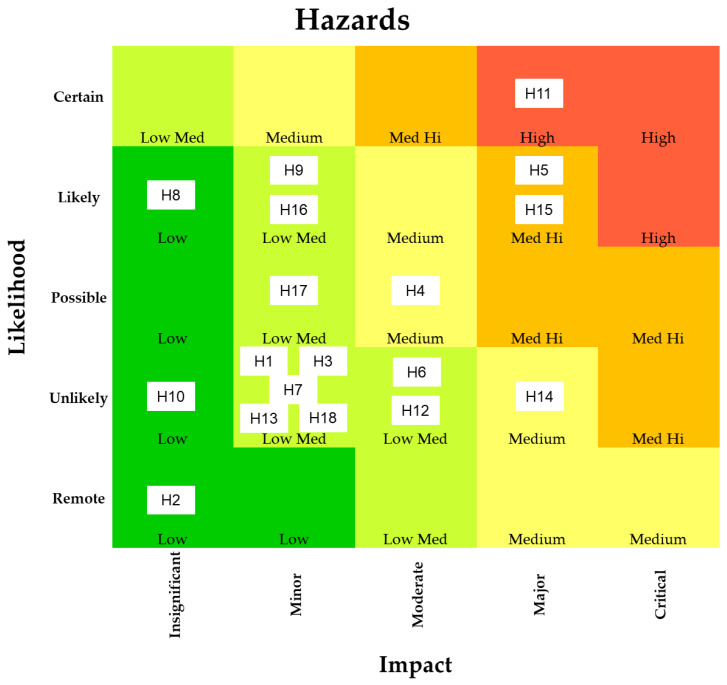
Hazard assessment taking eighteen possible hazardous compounds related to soy- and pea-based meat analogues into consideration.

To provide a better overview of the identified hazards presented in Figure 4, each of them is shown with a corresponding code. The definition of codes H1 to H18 is found in Table 3. 

Even though, in this hazard assessment, both the probability and the impact are considered, the validation, verification, and acceptance of the found results should be determined in a formal risk assessment.

### 5.1. Nutritional Aspects

Proteins are essential to the human health, since they can be digested into small peptides or free amino acids and be absorbed into the bloodstream for growth and maintenance of health. A decreased protein digestibility will lead to a reduction in bioavailability of such peptides or amino acids. Furthermore, proteins which are not fully digested can be utilized by gut microbiota to produce biogenic amines, which can lead to the development of bowel diseases [39,40,41,42]. 

Legumes and oilseeds, e.g., pea and soybeans, are nutritious foods containing carbohydrates, protein, dietary fiber, vitamins, and minerals. They can provide phytonutrients, vitamins, minerals, fiber, and ω-3 fats, as well as anti-nutritional factors (ANFs) like phytic acid, trypsin inhibitors, and tannins. Antinutrients are part of the defense mechanisms with which plants protect themselves from the surrounding environment. ANFs are usually divided according to their effect, e.g., trypsin and chymotrypsin inhibitors are known to influence protein digestion. Phytates and oxalates decrease mineral absorption, whereas saponins and amylase inhibitors affect the starch digestion [43]. Consequently, whole-plant foods are known to have, among others, lower digestibility and bioavailability compared to animal-based foods (e.g., egg, milk, muscle) due to the presence of ANFs and a cellulose cell wall that limits the access of enzymes for protein hydrolysis during digestion [44,45].

Phytates and oxalates are naturally occurring compounds found in many cereals and legumes. Since humans lack the enzyme phytase, they can pass unabsorbed through and bind to specific minerals, e.g., calcium, iron, and zinc, and reduce their absorption in the digestive tract [46]. Lectins are glycoproteins that interact with specific sugars in cell membranes, causing agglutination of red blood cells in-vitro, which stops the nutrient absorption during the intestinal digestion [47,48]. Polyphenols and tannins are astringent and bitter-tasting plant compounds which bind to proteins and minerals, reducing their bioavailability, inhibiting trypsin, alpha-amylase, and lipase enzymes. Saponins and their precursors are steroid compounds found in plants, where they form a waxy protective coating. Saponins may trigger the lysis of erythrocytes, which can deteriorate intestinal mucosal membranes, and bind to cholesterol and lipids [49].

Isoflavones are a type of polyphenol or phytoestrogen found in legumes and oilseeds like chickpeas, fava beans, and soybeans [50]. Consumption of foods containing isoflavones may increase the body’s level of antioxidants and help support cellular health [51]. However, the intake of phytoestrogen has also been related to have negative effects on the human health. Isoflavone compounds like daidzein, genistein, and glycitein have a similar structure to human estrogenic hormones such as 17-β-estradiol and display in-vitro estrogenic activity. Correlations between soy phytoestrogen intake and risk of, e.g., premature onset of puberty, breast cancer, hypospadias, and thyroid dysfunction have been found [52]. However, the findings were either inconsistent across studies or the number of studies was too low to draw confident conclusions. The content of isoflavones (H18) is affected to some extent by processing, with the highest levels being found in whole-bean products, such as tofu and cooked soybeans (3–4 mg isoflavones, expressed as glycones, per gram of protein). Processing conditions to obtain SPI and alcohol extracted SPC lead to a reduction of isoflavones to 1 mg and 0.2 mg per g protein, respectively [53]. Although isoflavones, when consumed at levels found in soy foods, can help maintain blood vessel health [54], they are only a small constituent of soybeans and thus soy protein ingredients. Therefore, their health benefits might be reduced when SPC- and SPI-containing products are consumed, since their concentration is lower. Additionally, extrusion of soy proteins might lead to a reduction of these compounds [50]. Although the total isoflavone content of soy protein concentrate mixtures was not reduced after extrusion processing, there was a reshuffling between the isoflavones leading to a lower effectiveness in preventing the proliferation of breast cancer cells in-vitro [51]. Although no data is available, in PBMAs, the concentration of isoflavones is expected to be even lower due to re-formulation. Therefore, the content and activity of these compounds in plant-based products might be so low, that neither positive nor negative effects arise after their consumption, making them a low-medium hazard.

It has been demonstrated that processes like soaking, germination, fermentation, pH-shifts, and thermal treatments reduce the trypsin inhibitor activity (TIA), tannins, and the antinutritional protein lectin found in legumes [44,55,56,57]. Even though ANFs can be lowered by adjusting milling and air classification process parameters, a combination of heat treatment and wet extraction will be the most effective process to reduce them [55,56,58]. 

The presence of dietary fiber and starch could also influence in a negative way the enzyme-catalyzed hydrolysis during protein digestion. It has been reported that the presence of dietary fiber reduced ileal protein digestibility in pigs [59]. Although starch, especially after heat treatment due to starch gelatinization, seems to influence more significantly the reduction of protein hydrolysis than fiber, it seems that addition of fiber can counteract this effect [56,60]. 

The nutritional value of proteins depends on the composition of essential amino acids (i.e., EAAs), bioavailability of proteins as a function of digestibility (i.e., speed and efficiency of digestion), processing conditions, and interaction with other compounds in the food matrix (e.g., ANFs, starch, or fiber). There are different ways to measure protein quality, e.g., the amino acid score (AAS) or protein digestibility corrected amino acid score (PDCAAS). PDCAAS is based on the essential amino acid requirements of preschool children (2–5 years), as published in 1985 [61]. In this measure, the AAS is corrected for the actual fecal digestibility of the test protein as measured in a rat assay. PDCAAS has been adopted as the standard method for measuring the value of proteins in the human diet [62]. The highest PDCAAS values are observed for proteins obtained from animal sources as expected. Compared to milk and egg proteins (PDCAAS value of 1.00), proteins obtained from beef have lower PDCAAS values (0.92).

Digestible indispensable amino acid score (DIAAS) is a more recent protein quality characteristic which was implemented to overcome issues regarding the overestimation of protein in products containing ANFs, not adequately accounting for amino acid bioavailability, and overestimation of the quality of poorly digestible proteins supplemented with limiting amino acids [63]. DIAAS uses ileal digestibility (ID) for each EAA rather than single fecal crude protein digestibility, i.e., determined at the terminal ileum at the end of the small intestine, which better reflects the absorption of each amino acid. For a given reference protein amino acid pattern (specific for toddles, children, adolescences, and adults), the DIAAS is the lowest calculated value (i.e., the EAA having the lowest digestible reference ratio). Pork meat, casein, egg, and potato proteins have excellent protein quality since they have an average DIAAS above 100. 

#### 5.1.1. Protein Quality

Soy is a high-quality source of protein that provides adequate amounts of essential amino acids to meet the needs for growth and repair of both children and adults. The values found in the literature related to protein quality are depicted in Table 4. Soy proteins have a good quality; however, for infants, methionine fortification is recommended to match breast milk. Although soy concentrates and isolates have PDCAAS values of 1.00 and DIAAS ≥ 75 [45,64], the gastric and intestinal in-vitro digestion of soy protein isolate leads to lower values of soluble nitrogen 74% and 66% compared to whey protein with 99% and 76%, respectively [65]. This lower value for soybean isolate could be attributed to the lower solubility of the gastric digestion products due to the presence of various disulfide bonds, which stabilize the protein structure, making it less susceptible to proteolysis [66]. However, if the stabilizing bonds are weakened, e.g., by thermal treatment, proteolysis would be facilitated. Moreover, heat treatment including toasting is often implemented during soy processing to inactivate enzymes and protease inhibitors and to improve off flavors [67]. Therefore, products that are less heat-processed, such as soymilk or tofu, have higher trypsin inhibitors than soy protein concentrates (i.e., SPC) and isolates (i.e., SPI). Oxalate can be present in soy protein products, which might influence the absorbability of calcium. However, when the absorbability of calcium from soy-based sources was compared with oxalate-free sources, such as cow’s milk, comparable values were obtained [68,69].

Similarly to soy, peas are also a major sustainable crop due to their nitrogen-fixation benefits and a source for global protein supply. Pea seeds have higher protein, total dietary fiber, and total sugar content compared to whole wheat. Protein content varies from 18% to 30% depending on environmental factors and the cultivar. Pea proteins have been widely consumed in Europe and North America as an alternative vegetable protein due to their nutraceutical attributes. They contain essential amino acids in adequate proportions, whereby methionine is the limiting amino acid in peas [77]. Although pea protein concentrate has a lower value than soy and other animal-based protein sources, its PDCAAS value is higher than other common plant proteins, like rice. However, gelatin, rapeseed, lupin, canola, corn, hemp, fava bean, oat, pea, and rice proteins were classified in the no quality claim category (DIAAS < 75) [45]. 

#### 5.1.2. Nutritional Value (H15) and Protein Metabolites (H16)

In general, meat products not only contain all the necessary amino acids, but also have a high protein digestibility. The DIAAS of bovine meat depends on the cooking conditions and ranges between 80 and 99%, as determined in pigs [78]. For plant-based intermediate meat analogues from soy and pea, it has been reported that the PDCAAS and IVPD are higher than 54%. However, the protein digestibility of the final product can differ from that of the intermediate product since further processing, including heat treatment during cooking and the use of additional ingredients in the formulation, might influence the digestion and absorption of nutrients [79]. Comparing soy- and pea-based beef or burgers with animal-based products, it is clear that the nutritional value (H15) of these products differs [39,73,75]. However, there are products like some soy-based burgers that are excellent protein sources with no limiting amino acids for individuals older than 3 years since the DIAAS of these products is above 100.

The digestibility in the stomach and small intestine of real beef and plant-based beef was compared and it was found that the total digestibility for beef was approx. 95% and for the plant-based product was 70% [73]. Additionally, it was reported that plant-based products were digested in the stomach phase more rapidly. However, the digestibility in the gastrointestinal phase was reported to be much lower: approx. 80% for meat and between 50 and 60% for the plant-based meat products [39]. The differences in digestibility between meat and plant-based meat products were discussed to occur from the different protein sources present, i.e., fibrous versus legume proteins. Moreover, it was observed that the lower digestibility could be related to an increase viscosity in both the gastro- and gastrointestinal phases. This increase in viscosity could result from the protein aggregation during, e.g., extraction and extrusion processing, or from the presence of dietary fiber in the formulation of plant-based meat products [39]. Correlated to the digestibility, it was found that more peptides were identified from the digested meat products compared to the plant-based analogues. Even though, beef might be a better option, both meat and plant-based meat analogues seem to be a good source of biologically active peptides [39].

The gut microbiome and metabolome can serve as indicators of health status. Accordingly, several metabolites (H16) with important regulatory roles in human health were found either exclusively or in greater quantities in beef samples than in the plant-based alternative and vice versa [80]. Nutrients found in meat, i.e., beef, include creatinine, hydroxyproline, anserine, glucosamine, and cysteamine, which potentially have important physiological, anti-inflammatory, and/or immunomodulatory roles. Low intakes of such nutrients can be associated with cardiovascular, neurocognitive, retinal, hepatic, skeletal muscle, and connective tissue dysfunction [81,82]. However, these nutrients are only found in beef. Phenols, tocopherols, spermidine, and phytosterols were found exclusively or in greater abundance in plant-based meat when compared to beef and are related to antioxidant, anti-inflammatory and/or cancer-protective properties; these, may benefit human health by dampening oxidative stress and inflammation, which might have potential neurocognitive and cardiovascular benefits [83,84,85,86]. 

Results from a randomized controlled trial to assess the changes in the gut microbiota of a group of 20 participants who replaced several meat-containing meals per week with meals cooked with plant-based meat products showed that consuming such products as part of a healthy balanced diet can elicit changes in the gut microbiota consistent with positive health outcomes [39,87]. Soluble and insoluble fiber and phytonutrients present in plant-based meat analogues have been reported to benefit human health [88], especially since they favor the growth of short-chain fatty acid metabolizing microbes. Therefore, the joint abundances of butyrate-producing microbes and the heightened presence of synthesis pathways in the intervention group could be interpreted as a positive sign resulting from the consumption of plant-based meat products, which is consistent with the microbial signature of “a healthy gut” [87]. 

To achieve the same nutritional value as animal-based products higher quantities of plant-based products might be needed. However, as this might also lead to more un-digested protein, the risk of developing bowel diseases might increase [41]. In the EU, the intake of protein with approx. 85 g/per capita/per day, is higher than recommended in the WHO guidelines (70% higher) [89]. Of the average protein consumed in the EU, approx. 35% is derived from plant-based proteins. Consequently, the decreased availability of nutrients in plant-based meat analogues represents a hazard for consumers whose diet is only based on plant-based foods and whose average protein consumption lies below the recommendation, as well as, for children aged below 3 years and the elderly. Therefore, the probability of this hazard (H15, nutritional value) was chosen to be likely, and the impact on health was considered major, making it a medium-high hazard.

However, in some regions in the world, the share of plant-based protein being consumed is already above the recommendations [90]. In these specific cases, the increase in undigested protein would be the hazard and not the decreased availability of nutrients.

Although it has been shown that the consumption of plant-based meat analogues might positively influence the gut microbiota, no long-term studies are available. Additionally, it is not clear if the potential benefits of metabolites obtained from meat analogues reduce the effect of lacking metabolites from animal-based products or how overall human health is influenced when solely these metabolites are consumed. Consequently, protein metabolites (H16) from PBMAs have a minor impact and thus can be considered as a low-medium hazard.

#### 5.1.3. Anti-Nutritional Factors (H17)

Dehulling, soaking, fermentation, germination, roasting, and other thermal treatments like boiling, or microwaving may be used to reduce anti-nutritional factors (H17) like phytic acid levels, trypsin and chymotrypsin inhibitors, and saponin levels, to partially or totally inactivate lipoxygenase, lectins, polyphenols, and tannins [77]. Therefore, it was shown that proteins extracted via dry-extraction, e.g., air-classified pea concentrate, contain higher amounts of antinutrients, e.g., plant phenolics (tannins), phytic acid, protease and trypsin inhibitors, and saponins, than ingredients from wet extraction, e.g., pea protein isolate [91,92,93] (Table 5). Changes in the protein conformation due to heat treatment may expose previously inaccessible proteolytic cleavage sites, leading to higher digestibility [94]. However, extensive heating of protein isolates might lead to a decrease in protein digestibility due to protein aggregation, which hinders protein hydrolysis by the digestive enzymes [56]. It seems that not only the degree of denaturation, but also the protein size and conformational states, influence the protein digestibility. 

High temperatures are known to reduce the nutritional quality of food products. However, extrusion cooking has been related to be the chosen processing technique for high productivity and significant nutrient retention due to a high temperature-short time treatment [95]. Additionally, the mechanical forces acting on the proteins due to the rotation of the screws are expected to break protein bonds that stabilize its molecular structure, such as covalent bonds. Moreover, it has been reported that extrusion temperatures higher than 140 °C and feed moistures ranging from 12 to 25% would destroy trypsin inhibitors almost completely in legume products [96,97,98]. Consequently, extrusion processing might lead to a further increase in, e.g., IVPD. However, this also depends on the protein source and processing conditions.

**Table 5:** Comparison of anti-nutritional factors for soybeans and peas as raw materials, as protein concentrates (soy protein concentrate (SPC) and pea protein concentrate (PPC)), as isolates (soy protein isolate (SPI) and pea protein isolate (PPI)), and as extruded samples. Data extracted from references [77,99,100,101,102,103].

Parameter	Soy	Pea	SPC or SPI	PPC or PPI	Extruded Soy	LME Pea	HME Pea
Phytic acid/%	1.32	0.54–1.2	1.1–2	0.46–1.6	–	–	–
Trypsin inhibitor/TIU/g	49,450	2100	110–2110	1300–2300	190–1480	378–1410	168
Lectin/g/kg	5.88	5.77	<0.1	5.98–23.2	~0	–	–
Tannin/%	0.46	1.12	–	0.47–1.79	–	–	–

The reduction of trypsin inhibitors increases with extrusion temperature, moisture content and residence time. 89% reduction of trypsin inhibitors was achieved for soy flour extruded with a barrel temperature of 153 °C, 20% moisture and 2 min residence. In addition to the trypsin inhibitor, lectin and urease activities were almost completely lost after the extrusion of soybeans [104]. Low and high moisture extrusion of pea proteins led to a significant reduction of 82% and 92% in trypsin and chymotrypsin inhibitor content, respectively, which indicates that the reduction of trypsin inhibitors increases with moisture content [98,103]. Furthermore, the protein digestibility of soybeans by pepsin and trypsin increased after the extrusion process, probably due to denaturation of proteins, rendering them more susceptible to pepsin activity [105]. Similarly, an increase in protein digestibility was observed for legume seeds after extrusion processing [106].

In addition to soy, extrusion processing leads to reduction or complete elimination of lectin activity in legume flours [96,106,107]. The IVPD of pea flour increased from 81% to 90% when extruded at 90 °C [108]. The mean amino acid digestibility of extruded yellow pea protein was approx. 88% [109]. Lower values of PDCAAS and DIAAS were obtained for extruded yellow pea, 74 and 70%, respectively. In contrast to extrusion processing, cooking of peas led to lower values ranging between 67 and 69% [71]. Increasing the moisture content of texturized pea products led to an increase in available lysine but a decrease in protein digestibility. Similar to soybeans, increasing the temperature and screw speed led to higher values of protein digestibility but less available lysine for extruded pea and soy protein mixtures [110,111]. The IVPD of low moisture extruded pea concentrate and soy proteins was reported to be approx. 96% [58]. However, lower values of 54 and 64% were also reported for extrusion of pea protein isolate using high- and low-moisture conditions, respectively [112]. Moreover, it has also been reported that extrusion at higher moisture contents of pea protein isolates had no effect on amino acid composition or degree of hydrolysis indicating that high shear and temperature, short time and high moisture did not cause amino acid losses or formation of peptide bonds [113]. Similarly, it was reported that the protein digestibility of lupin protein did not change significantly (>80%) at 55% moisture content with temperature (155 and 180 °C) and screw speed (400, 1200, and 1800 rpm). However, the highest IVPD was observed at 155 °C and 68% moisture, indicating that milder conditions for protein aggregation lead to higher digestibility [114]. In general, it must be pointed out, that the values for the extruded isolates were higher than those of the non-extruded protein. 

Furthermore, extrusion processing of various legumes flours, including pea, led to a redistribution of soluble/insoluble dietary fiber, which might influence the protein digestibility [115]. Although the mineral content of peas did not significantly change after extrusion processing, an increase in the absorption of iron, copper, and phosphorus was observed probably due to a reduction in ANFs [116]. Protein digestibility can be compromised by the formation of protein aggregates via hydrogen bonds, hydrophobic interactions, and disulfide bonds, with a consequent decrease in solubility. Defatted soybean concentrates (approx. 60% protein) that were extruded at temperatures of >240 °C and low moisture (10%) contained lysinoalanine and lanthionine, which are cross-linked proteins not available in nature that cannot be digested [117]. Even though these compounds were also present in the protein concentrates, it is known that extrusion processing of proteins at high temperatures and low moisture contents can lead to the formation of undesirable compounds. Moreover, Maillard complexes may also be formed at such conditions. The Maillard reaction particularly affects the bioavailability of lysine, and probably also of arginine, tryptophan, cysteine, and histidine [118]

Commercially available plant-based meat analogues are burgers, nuggets, meatballs, cold-cuts including plant-based mortadella, and whole-cuts including plant-based steaks. The nutritional composition of these products varies and further preparations like frying the nuggets affect the final composition [119]. In the final formulation, the addition of different ingredients like fibers and starches further influences the protein digestibility, so it can be higher or lower than after extrusion processing. However, no relevant studies were found, where the ANFs were determined in the final formulation, probably because the overall nutritional value, i.e., protein quality, already takes the effect of ANFs indirectly into consideration. Since the ANFs affect the nutritional value and the protein digestibility, they are categorized as a low-medium hazard due to the fact that the overall nutritional value already indirectly takes the effect of ANFs into consideration.

### 5.2. Allergens (H11)

Proteins can induce allergic reactions in humans. Most of the proteins that induce an allergic reaction in humans have similar structural features, e.g., multiple, linear immunoglobulin-E (i.e., IgE) binding epitopes, multiple disulfide bonds, and glycosylation. Even though they have similarities, it is not possible to identify a conformational sequence pattern common to all allergenic proteins [120]. However, it is known that many of the plant food proteins that trigger an allergic response through the gastrointestinal tract belong to either the cupin family, i.e., vicilins and legumins, or the prolamin family [121]. Soy is one of the nine major food allergens besides milk, eggs, fish, Crustacean shellfish, tree nuts, peanuts, wheat, and sesame. At least 30 allergens, including profilin, vicilin (i.e., β-conglycinin), and legumin (i.e., Glycinin), have been identified in soybeans [122]. β-conglycinin and glycinin represent approx. 70% of the proteins in soybean and therefore will be the predominant proteins in commercial soy protein products [123,124]. Processing can reduce soybean allergenicity since it can change the proteins at their molecular level. Among others, thermal processing, enzymatic hydrolysis, and fermentation can reduce the allergenicity of soybean protein by destroying the epitopes as a result of protein degradation [122,124]. Even though the incidence of soy protein allergy is much lower (0.4%) than e.g., milk proteins (2.5%) [125], they do induce food allergy reactions. Pea proteins may also be a potential food allergen [126,127]. Similar to soy protein, green pea allergenicity is attributed to the legumin and vicilin fractions [127]. The convicilin and vicilin fractions of crude pea extracts were found to induce an allergic reaction in more than 50% of the individuals in the panel [126]. However, germination seemed to reduce the allergenicity of soybeans and peas [128]. Similarly, other processing conditions like thermal and enzymatic treatment are expected to reduce the allergenic potential of pea products. Accordingly, blanching can reduce at least some pea-allergic individuals [127]. Moreover, the results indicated that the allergenicity of pea hydrolysates decreased significantly after enzymolysis, and the allergenicity of ultrafiltrated components was significantly lower than that of other isolated and purified components [129].

It has been reported that processing conditions during the production of plant-based meat analogues can lead to a reduction in the allergenic potential of plant proteins [103,122,124,130,131]. However, there are some studies suggesting that it might increase [132]. The effect of processing conditions, including thermal treatment, on the allergenicity is related to either the masking/destruction of epitopes or their exposure or generation of new ones, resulting in their decrease or increase, respectively [133,134,135,136,137]. Extrusion processing can be used to reduce the allergenic potential of novel protein sources. High and low moisture extrusion significantly reduced the globulin and β-conglycinin content (>49%) of pea and soy proteins [103,131]. Accordingly, it was reported that temperatures ≥ 140 °C during extrusion processing would destroy the epitopes of β-conglycinin completely and consequently significantly reduce the antigenicity of soy [138]. Extrusion processing also leads to a reduction in the immunoreactivity of SPI/corn and soy flour/corn mixtures at temperature above 110 °C and moisture content > 20% [139]. Extrusion processing can, therefore, lead to a decrease in the allergenicity of plant-based proteins, depending on the processing conditions and the raw materials. In contrast, it can be said that if during the processing of plant-based proteins Maillard reactions are expected, then neoantigens such as advanced glycation end products (AGE) can be formed, which would increase the allergenicity of the processed proteins compared to non-processed proteins [140]. Since no process is known that can completely eliminate possible allergens at present, extreme caution is needed as cross-reactivity between allergens might induce allergic reactions related to products that were not known to be allergenic to consumers. For example, among pea proteins, convicilin has been shown to interact with lentil vicilin and induce an allergenic response [126]. However, data related to the allergenicity of the final products consumed, i.e., plant-based meat analogs with a complex formulation, is not available. Therefore, until data is available, the potential allergenicity of these products seems to be a hazard with high probability of occurrence and with a major impact, making it a hazard with a high score. This parameter needs to be extensively studied through further in-vivo clinical investigations [141]. These results are necessary to (re)-define regulations related to the labelling of plant-based meat analogues.

### 5.3. Microbiological Aspects

Several biological contaminants are related to plant-based proteins. Biological contaminants include bacteria, viruses, and parasites. The contaminants present in products, which are consumed by humans, should be controlled, since they may lead to outbreaks of foodborne illness and even deaths. A low medium hazard unlikely to occur but with a moderate impact is the presence of spore- and non-spore-forming bacteria (H12).

Millions of infections annually originate from foodborne pathogens such as *Campylobacter* spp., *Salmonella* spp., and *Escherichia coli*. These pathogens are transferred to humans among others with animal-based products. Even though some approaches including vaccination of animals are implemented worldwide, the number of caused diseases is not decreasing. In contrast, since the human-animal interaction is eliminated, the risk of zoonoses and infectious diseases from plant-based meat analogues decreases [8]. 

Contamination of plant-based products can result from contact with soil, dust, and water during harvest and post-harvest periods [142,143]. Additionally, processing may introduce contaminants like *Staphylococcus aureus*, mainly via food handling (contact with skin), or *Listeria monocytogenes*, as it can be in the factory environment. Moreover, viruses may be introduced via factory workers. However, processing steps like heating may also lead to reduction of these contaminants. Consequently, roasting and mild heat treatment have been used to preserve the properties of various crops. Nevertheless, some pathogens, such as *S. aureus*, can remain even after mild heat treatment and continue to grow during storage and transport [143]. West-African soy was evaluated, and bacterial contamination and presence of enteric pathogens was found [144]. Furthermore, various biological contaminants, including *Salmonella* spp., *B. cereus*, *C. perfringens*, *S. aureus*, *C. jejuni*, *C. botulinum*, STEC, norovirus, and the parasite *Cyclospora cayetanensis*, have been found in cereal products and legumes including peas and beans [145,146,147,148]. Fungi with the ability to produce toxins including *Penicillium* spp., *Aspergillus ochraceus*, *Aspergillus niger*, and *Aspergillus glaucus* have been found in peas [149,150]. Consequently, *C. botulinum*, *C. perfingens*, and *B. cereus* are spore-forming bacteria identified as microbiological hazards related to beans by the FDA [151]. Even though heat treatment might lead to the inactivation of pathogens, the processing conditions have to be adequate. 

Dried and powdery products like plant-based protein powders are considered safe since their dry state and low water activity prevents microbial development and may maintain populations at undetectable levels [152]. However, the actual biological load depends on the processing steps to manufacture these ingredients. If the raw materials such as peas and beans are contaminated and the protein extraction method, e.g., dry fractionation, does not include a heating step, the biological pathogens might not be inactivated. Therefore, they could be present in high enough concentrations in the protein powders to cause a risk to human health due to potential foodborne illnesses. Additionally, if pesticides or other chemical compounds are used to hinder their growth, their residues may end up in the raw materials, and, consequently also in the protein ingredients, which could present a hazard from a chemical point of view, as will be discussed in the next section. Various studies, published and unpublished, show that plant-based protein ingredients can contain significant levels of microbial contaminants, including the spore forming bacteria *B. cereus*, *B. amyloliquefaciens*, and *Geobacillus stearothermophilus*. Additionally, *B. subtilis* and *B. licheniformis* have been found in plant-based protein powders [153,154]. Some of these contaminants can produce heat-stable toxins and/or heat-resistant spores that can survive even high heat treatments and can also contain low concentrations of heat-resistant molds [153,154]. However, there are also studies pointing out that various plant protein powders have a low microbial load, implying that they are safe for consumption [155,156]. Therefore, each ingredient has to be considered separately and the biological contaminants should be measured and evaluated regularly as is done during the quality control. 

Extrusion processing conditions to produce meat analogues include water contents between 20 and 80%, temperature above 90 °C, pressures > 1 bar, and residence times up to minutes. Therefore, it is expected that microbial cell and bacterial endospores are almost or completely inactivated [157]. Extrusion processing of soy flours resulted in very low amounts of *Staphylococci*, *Salmonella*, *Shigella*, yeasts, and molds. Moreover, extrusion processing also led to reduction in the total plate count and number of *E. coli* [158,159]. It has been reported that extrusion can reduce the viability of unicellular organisms due to thermal processing. Additionally, the shear stresses acting on the melt during extrusion may reduce the microbial load, suggesting that mechanical forces can lead to cell rupture [160,161]. Intermediate meat analogues produced with pea and soy protein showed no microorganisms including *Enterobacteriaceae*, pathogenic bacteria like *Listeria monocytogena* or *Salmonella* spp., *Bacillus* spp., *Staphylococcus aureus*, yeast, molds, *P. aeruginosa*, and psychotrophic microorganisms. No pathogenic bacteria including *Staphylococci*, *Salmonella*, *Shigella*, or *E. coli* were determined after extrusion processing [156,157]. Although contamination of the protein isolates was slightly over the detection limit, the extrusion step eliminated them completely [156]. The microbiota of the extruded products did not contain any Gram-negative bacteria and very low concentrations of Gram-positive bacteria (<<100 CFU/g). However, during further processing and reformulation, (re)-contamination was observed, which was the case for some vegan and vegetarian products since *Enterobacteriaceae* were found [152,162,163]. Accordingly, in different plant-based meat analogues, including vegetable sausage, some pathogenic bacteria, e.g., *Acinetobacter*, *Staphylococcus*, and *Klebsiella*, were detected [164]. 

On the other hand, the products that were thermally processed after packaging, i.e., pasteurized or sterilized, were microbiologically stable for several weeks [152,157]. The shelf life of plant-based meat analogues can be similar to meat products or longer if preservatives or further thermal processing takes place after packaging [157]. The microbial spoilage (H13) of chicken and vegan rolls was compared, and no differences were revealed. However, the samples were only acceptable for up to 12 days of storage at 4 °C and up to 3 days of storage at ambient temperature [155]. Therefore, when the cooling chain is not hindered, and the storage and consumption recommendations of the manufacturers are followed, no significant hazard should arise, making this a hazard with a low-medium score.

#### Mycotoxins (H14)

A variety of fungi (molds) species such as *Fusarium*, *Aspergillus*, *Penicillium*, *Alternaria*, and *Claviceps* can synthesize mycotoxins as secondary metabolites [165]. 

Over 100 fungal species have been found to produce a variety of mycotoxins, of which trichothecenes, ochratoxins, aflatoxins, zearalenone, fumonisins, patulin, and citrinin are the most toxigenic to, e.g., public health [165]. 

Grain and crops, including legumes, have been shown to be occasionally contaminated with mycotoxins (H14). Among others, ochratoxin A and zearalenone were found in soybeans, soy-based products including shoyu and miso, and peas [150,166,167,168,169,170,171,172]. Although it was not reported, it is expected that if soybeans or peas are contaminated with mycotoxins, the flours, and consequently the protein powders, will also be. However, depending on the extraction process, the degree of contamination might change. Washing and soaking steps can reduce up to 50% of the mycotoxins present in the raw materials since they accumulate in the water used for each step [173]. But these contaminants can be still present at significant concentrations in the protein powders and the exact concentration depends on the extraction process [174]. Extrusion of various cereals led to significant reduction of fumonisin, aflatoxins, and zearalenone (>83%), while the reduction of deoxynivalenol, ochratoxin A, and moniliformin was not as significant (30–55%) [175,176,177]. Additionally, extrusion combined with ammonium hydroxide led to a significant reduction of these contaminants [175,176,177]. Still, no studies regarding the presence of mycotoxins in plant-based meat analogues are available. In this case, mycotoxin occurrence is possible, and its effect on human health is critical. However, manufacturers are obliged to assess for significant contaminants, such as mycotoxins, as part of the quality control and management system. Therefore, since their presence is controlled, they are unlikely to be present. But their impact is major, which makes it a hazard with a medium score. 

### 5.4. Chemical Aspects

Pesticide residues have been found in grains, e.g., chickpea, at quantities exceeding the maximum residue limit right from the beginning of storage. However, processing techniques such as soaking and germination eliminated almost all the pesticide residues found in the grains [178]. Legumes like fava beans, may contain chemical compounds like glycoalkaloids, which cannot be degraded during digestion, since humans lack the corresponding enzyme, i.e., G6PD. This can lead to favism, which is a disease that causes acute hemolysis. In 2018, a nonprofit group called the Clean Label Project released a report about protein powders including plant-based ingredients, which contained pesticides, heavy metals (e.g., lead, arsenic, cadmium, and mercury), bisphenol-A (i.e., BPA), and other contaminants linked to cancer and other health conditions. Moreover, one protein powder contained 25 times the allowed limit of BPA [174]. Hexane (H2) is a solvent used to extract and separate vegetable oil from almost all oilseeds, including soybean, canola, sunflower, and olive oil. For soybeans, it is used during the separation of soy oil and soy meal in the crushing stage, and it can lead to neurotoxicity, when high concentrations of its vapor are inhaled. However, the residual levels of hexane found in soy products including SPI are typically less than 1 ppm. In the EU hexane is permitted for production of defatted flours and the residue limit is 10 ppm in the food product manufactured from those flour products [53]. Rat feeding studies showed that 400 ppm/day for 28 days did not lead to adverse reactions [179]. Therefore, assuming that no hexane evaporates during cooking, it would take various hundreds and even thousands of kilograms of plant-protein based products in a daily basis to reach that level making it a remote, low hazard. Further contaminants with toxicological properties such as chloropropanols can be found in some vegetable protein powders. Chloropropanols (H3) are formed during acid-mediated hydrolysis for the manufacturing of vegetable products, when the acid reacts with residual lipids present in defatted meal from e.g., oilseeds or with vegetable proteins in e.g., maize, wheat, and rice. The maximum level of 3–monochloropropane–1,2–diol in food products has been established to be 0.05 mg/kg in the dry matter [180]. Since heavy metals (H1) are also commonly found in nature, including in soil, they can also be present in many foods, including grains, crops, and proteins extracted thereof. Even though there may be some detectable levels of heavy metals in some plant proteins, their concentration in the final product is even lower. For chemicals (H1 and H3) to be a hazard for the human health, chronic or high-level exposure is needed. Therefore, these compounds have a low-medium score.

Another substance likely to be present in plant-based meat analogues is methylcellulose (H8). The presence of methylcellulose in plant-based products is a low-score hazard since even though it is likely to be present in plant-based meat analogues, its impact on human health is not significant. This substance is a modified cellulose (dietary fiber) added to food formulations as a binder [181] and is known to affect glucose metabolism similarly to other dietary fibers. During glucose tolerance tests, the addition of different dietary fibers was tested. It was found that their addition can lead to a reduction of blood glucose concentration and serum insulin concentration [182]. In 2018, the use of unmodified and modified celluloses as a food additive was concluded to be of no safety concern by the EFSA [183]. Carrageenans (H9) are incorporated in various food products due to their thickening and gelling properties. A fraction of carrageenan formed through acid hydrolysis and extensive heating has been reported to cause, e.g., intestinal ulceration [184,185]. The yield of this fraction has been found in the residues after gastric digestion of carrageenan to be approx. 10%, which could be enough to cause adverse reactions in humans [186]. However, there is a lack of studies to support or refuse these claims [187] making the presence of carrageenan a hazard likely to occur but with a low-medium score.

Furthermore, leghemoglobin (H10), a low hazard, is present in certain plant-based meat products to impart a meat-like flavor profile. Its safety has been questioned since its consumption could be related to an increase in body iron storage and therefore to an increased risk of developing type-2 diabetes [188]. However, in-vivo and in-vitro studies in rats reported no adverse effects, suggesting that there is no toxicological concern related to this ingredient [189]; therefore, it does not impact human health after consumption.

#### Process-Related by Products (H4–H7)

Some of the most intensively investigated issues in relation to meat consumption and health aspects for unprocessed meat are related to the overconsumption of meat and its high saturated fat contents. In contrast, for processed meat, the processing conditions, e.g., temperature and addition of salt, nitrite, and nitrate, are thought to be the most significant influencing factors since they affect the formation of carcinogenic compounds such as heterocyclic aromatic amine, acrylamide, N-nitrosamine, and polycyclic aromatic hydrocarbons [32,33,190]. These carcinogenic compounds can be formed when proteins are heated above certain temperatures, when nitrogen compounds and amines react, or when Maillard reactions take place, respectively. Therefore, these toxic chemical compounds may be also formed during the extraction processes, during extrusion to produce intermediate meat analogues or during further processing and re-formulation to obtain the final products. 

N-nitrosamines (NNAs) result from the reaction between a nitrosating agent, originating from nitrite, nitrate or smoke and a secondary amine, derived from protein [32]. Nitrite is converted to nitrous acid in acidic conditions, which is decomposed into nitrous anhydride (N_2_O_3_). Nitrous anhydride further reacts with amines to generate NNA.

Their formation can occur only under certain conditions, including strongly acidic conditions such as those in the human stomach. Additionally, high temperatures, as in frying, can enhance the formation of NNAs. Considering the process chain of, e.g., soy, during the extraction process, only mild thermal treatment takes place, which should not lead to the formation of NNAs. Even though, during isoelectric precipitation, the pH is lowered since no nitrosating agent is added or present, no NNA formation is expected. In the second processing step, i.e., extrusion process, the extracted proteins are subjected to high thermal treatment (80–180 °C). In this case, the temperature might be adequate for the formation of this side product, however the nitrosating agent is still lacking, so NNAs are also not expected. In the final product, i.e., soy-based meatballs, vitamin B1 is added as thiamine mononitrate [3]. If these products are heated at very high temperatures, NNAs can be formed. NNAs were determined in commercially available meat analogue products after cooking under recommended conditions and most of them had undetectable contents of these chemical contaminants. However, NNA was detected in one cooked sample (approx. 15 μg/kg, data unpublished) [38], which is above the maximum level in processed meat set by WHO (10 µg/kg), and therefore unacceptable. However, no information regarding the sample, its type, or its manufacturers is available. Furthermore, NNA formation can be inhibited by the addition of antioxidants such as ascorbic acid and vitamin E, which are often added to the formulation of plant-based meat analogues. This might be the reason why the majority of the investigated products had undetectable amount of NNAs. 

Maillard reaction is a non-enzymatic browning reaction that occurs in foods during thermal processing e.g., baking or frying [191], leading to the formation of desirable color, flavor, and aroma. Heterocyclic aromatic amines (HAAs) are mainly formed during heating processes through Maillard reactions. Their formation is influenced by various factors, including cooking method and time, pH, lipid and sugar content, and concentration of free amino acids or even antioxidants [192,193,194]. Additionally, it has been observed that the amount of fat negatively influences their formation [195]. Polar HAAs are formed from amino acids, carbohydrates, and creatinine. Creatinine is especially necessary since the imidazo moiety is formed from this compound. If it is not present, no polar HAAs are formed. At higher temperatures, non-polar HAAs are preferably formed. They are usually assigned as pyrolysis products of the amino acids [196]. For non-polar HAAs, the formation of carbolines, including norharman and harman, has been reported [196]. Tryptophan is the main precursor and glucose facilitates their formation [197]. It has been reported that polar HAAs can be produced at temperature ranging between 100 and 300 °C [198]. However, creatinine is needed in the last step of the reaction. Since plant proteins do not contain creatine which can be transformed to creatinine by e.g., heat, the formation of this type of HAAs along the processing chain is not expected. However, the formation of carbolines might be relevant during the last processing step before consumption, as it has been reported that these compounds can be formed at even 40 °C in presence of, e.g., iron, which is often included in the formation of, e.g., burger patties. Nevertheless, no information is available for plant-based ingredients or PBMAs. Therefore, these compounds (H4), i.e., NNAs and HAAs, are found to be medium hazards.

Acrylamide, i.e., AA, is formed in a wide variety of foods, especially when carbohydrate-rich foods are cooked above 120 °C during frying, baking, and roasting [199]. Similarly, AA is formed when the free amino acid (asparagine) and reducing sugars (mainly glucose and fructose) react during the Maillard reaction [200]. Additionally, in the absence of asparagine, acrolein and ammonia can form AA in lipid-rich foods. It is known that acrolein and acrylic acid are produced by the degradation of lipids (triglycerides) subjected to high temperatures [201,202]. Degradation of amino acids with ammonia can give rise to acrylamide formation via thermal decomposition [203,204]. Amino acids such as glutamine, cysteine and aspartic acid have also been found to produce low amounts of acrylamide [205]. Although there is no consistent epidemiological evidence on the effect of acrylamide from food consumption on cancer in humans, both the U.S. National Toxicology Program and the Joint Food and Agriculture Organization/World Health Organization Expert Committee on Food Additives (JECFA) consider acrylamide to be a human health concern. The content of AA in foods is not yet regulated; however, the benchmark level ranges between 40 and 850 µg/kg. Baby foods or food for infants and young children show the lowest level, whereas instant coffee shows the highest [206]. The AA content of various plant-based ingredients, including some TVPs, obtained via extrusion processing was recently investigated [207]. Even though the values depended on the protein source, i.e., pea, soy, or wheat gluten, the protein flours (less processing) showed lower values of AA compared to wet-extracted proteins. Surprisingly, TVPs had similar values to dry-extracted proteins. However, since the supplier differed, no correlations between protein source, process, and AA content could be obtained. The AA content for protein ingredients related to PBMAs ranged between 185 and 748 µg/kg [207]. Lastly, the AA content in the final products e.g., plant-based burgers was evaluated and the highest AA content of approx. 72 µg/kg was obtained for soy-flour based burgers fried at 200 °C for 5 min [208]. Before frying, a pea-based burger showed the highest values of AA at approx. 23 µg/kg, which did not increase during frying. A soy-protein burger had the lowest values of AA when unprepared and even after frying at 200 °C, the content of AA was lower than 20 µg/kg. As expected, the AA content increased with browning of the burgers and saccharide content. No AA could be measured for the ground beef burgers evaluated under the same frying conditions. Information about the manufacturers was available; however, the extraction conditions of the proteins used in the formulation is not known. Additionally, it is not clear if the proteins were further processed as in extrusion processing. Consequently, no correlation between protein source, extraction, and extrusion process can be made since the available information considers different products at different stages of the processing chain. Overall, AA was found in protein ingredients used in PBMAs. However, the content of the proteins in the final products e.g., burgers, is 20–30%; the AA content decreases from up 748 µg/kg in the protein ingredients to 72 µg/kg in the final products depending on the exact formulation. To date, there have been limitations regarding the AA content or even a maximum benchmark level for meat analogues. Consequently, the impact of AA was chosen to be major due to the fact that it is considered a health concern, but no epidemiological evidence exists and there are not regulations yet. The probability was chosen to be likely since it was found in AA. Therefore, AA (H5) was found to be a medium-high hazard.

Polycyclic aromatic hydrocarbons (H6), i.e., PAHs, are produced by incomplete combustion or high-temperature degradation of organic substances including proteins and lipids [209]. Hardwood or charcoal used as heat sources during e.g., grilling, will release low-molecular-weight PAHs when heated at high temperatures during the unstable primary combustion stage. Fats and oils, when used as cooking media might also release them under similar conditions [210]. Additionally, direct and indirect contact between food and heat sources (smoke, emissions, and fumes) can also cause PAHs to accumulate on its surface [190]. Nutrients such as fats, proteins, and carbohydrates are cracked at temperatures > 200 °C to produce low-molecular-weight light rings and intermediate free radicals with high reactivity [210]. The two are then cyclized and recombined to form high-molecular-weight polyamides [211]. The formed high-molecular-weight PAHs will gradually migrate to the hydrophobic part of the food and accumulate [190]. Considering the toxicity of PAHs, several countries have drafted regulations to establish tolerable limits for PAHs in food products and beverages. The current European legislation provides specific parameters for many food products including smoked meat and meat products. Maximum levels for PAHs (BaP and PAH4 of 10.0 and 50.0 µg/kg, respectively) in plant-based powders used for beverage preparations are regulated [212]. However, no evaluation of the PAH contamination in PBMAs or related plant-based ingredients is available. The limit of PAHs for plant-based powders is probably related to the PAHs present in the soil or other environmental pollutants and drying process after harvest to prevent spoilage during storage. Since they are formed at high temperatures (>200 °C), it is not highly probable that they are formed during extraction and extrusion processes. During LME to produce TVP, high temperatures are achieved; however, the residence time is short (<3 min). However, there are no studies corroborating this assumption. The post-processing step before consumption seems to be relevant since, in addition to ingredients in the formulation, i.e., lipid content, cooking techniques such as roasting, frying, and grilling contribute to PAHs’ formation in foods [213]. Since these compounds are unlikely to be formed when cooking at temperatures < 200 °C but no regulations exist for their occurrence in PBMAs, they represent a low-medium hazard.

Protein cross-linking via the dehydroalanine (DHA) pathway can occur in food protein systems. It is induced by heat treatment and/or alkaline conditions. In the first stage, a serine (including phosphoserine and glycoserine), cysteine, or a cystine forms DHA through a β-elimination reaction [214]. DHA further reacts via a Michael addition with either a lysine, a cysteine or a histidine residue, which are found in proteins and peptides, to form protein cross-links lysinoalanine (LAL), lanthionine (LAN), and histidinoalanine, respectively [215]. Lysinoalanine is present in a wide range of foods including dairy products, chicken, and sausages [216]. The cooking temperature and consequently cooking method influences the LAL content as it increases when the product is either cooked, charcoal-broiled, or microwaved (100, 150, and 200 µg/g protein, respectively). Covalent protein cross-links such as LAL have been already identified in soy proteins [217]. During the extraction process, the soy flour is subjected to high pH values and mild to high temperatures which can lead to the formation of these compounds. Alkali-treated soy flour and soy isolate was reported to contain 0.57 and 0.8 g of LAL/16 g of N, respectively [218]. LAL formation was also confirmed when soy proteins were subjected to alkaline conditions (pH 8–14) for different times (up to 48 min) and temperatures (25–95 °C) [219]. In contrast, not much information is available for LAN. Regarding the formation of this kind of covalent protein crosslink during extrusion processing, some indications can be found in the literature. The DHA and LAN content of wheat-gluten extrudates was evaluated and it was found that, with the addition of alkali, the cysteine level decreases and, thus, the DHA and LAN content increased [220]. Since pea proteins are rich in lysine and not so rich in cysteine, it is expected that LAL would be formed instead of LAN. Similarly, soy is not rich in cysteine; therefore, LAL is rather produced. However, no information is available on how these values change after further processing, i.e., extrusion processing and cooking, before consumption. There is not much information on how these compounds affect the human health besides that they reduce the protein digestibility and thus the nutritional value of food. Moreover, LAL was shown to induce nephrocytomegaly in rat kidney tissues after consumption [221]. The presence of these protein crosslinks is problematic and the safety implications for humans is still unresolved [214]. Therefore, LAN and LAL (H7) are a hazard with a low-medium score.

## 6. Concluding Remarks

This study reviewed potential hazards related to the production of plant-based meat analogues and their consumption. The variation of or introduction of new possible hazards along the processing chain from the extraction of plant-based proteins to the re-formulation of ready-to-eat meat analogues was discussed and it was found that even though some hazards remain similar to meat products, some new hazards arise. 

Overall, most of the discussed hazards have low to medium scores. Low hazards, including hexane (H2), a compound related to protein extraction, methylcellulose (H8) and leghemoglobin (H10), which are used a binders and flavoring, do not represent a particular case. Hazards in the low-medium hazard category include the presence of chemicals, i.e., heavy metals (H1) and chloropropanol (H3), compounds related to processing like polycyclic aromatic hydrocarbons (H6) and lysinoalanine and lanthionine (H7), carrageenan added in the final formulation as a binder (H9), spore- and non-spore forming bacteria (H12), spoilage organisms (H13), protein metabolites (H16), anti-nutritional factors (H17), and isoflavones (H18). Microbiological hazards (H12 and H13) are defined within the framework of self-monitoring in the hazard analysis and critical control points system (HACCP) of manufacturing companies. Therefore, provided that this internal process and regulations are followed, they do not represent a particular hazard. 

Various unknown variables are not fully described or analyzed and represent potential hazards to the human health. The presence of processing-related hazards such as N-nitrosamines and heterocyclic aromatic amines (H4) and acrylamide (H5) in plant-based products is often unknown and their concentration remains mostly unquantified, which makes them medium and medium-high hazards, respectively. Another medium-high hazard is the lower nutritional value (H15) of PBMAs compared to animal-based products. Soy- and pea-based beef or burgers had a lower nutritional value (H15), i.e., total digestibility, compared to animal-based products. Nevertheless, there are some products, like soy-based burgers, that are excellent protein sources with no limiting amino acids for individuals older than 3 years. The different nutritional value of these products suggests that they cannot be compared to, e.g., animal-based products. They are different products with their own positive and negative aspects, which should be taken into consideration when consumed. Moreover, it is not known whether the sole absorption of metabolites (H16) derived from plant-based products compared with animal-based products ultimately affects human health. Since these aspects are relevant for the risk assessment of plant-based meat analogues, they should be further investigated and analyzed. 

Allergens (H11) are a hazard with a high score since they are present in plant-based meat analogues and cannot be completely eliminated. Therefore, they present a threat to individuals prone to allergic reactions and their consumption should be carefully evaluated or avoided. 

In general, it was observed that most of the contaminants came from the raw materials or due to contamination during processing. Therefore, the use of high-quality ingredients and optimal choice of processing parameters would lead to safer products. Ultimately, there is not a general answer to: does the consumption of meat analogues have a positive or negative effect on the human health? This is because the positive and negative effects discussed in this review depend on many parameters, including protein source, processing, final product, and how the consumers include these products in their diets. Incorporating meat analogues into a balanced diet may positively influence gut microbiota and overall health outcomes. Nonetheless, to mitigate certain limitations these kinds of products might have, a diverse diet emphasizing whole foods is recommended; this is applicable not only to meat analogue-based diets but also to omnivorous and vegetarian diets.

## Figures and Tables

**Table 1:** Definition of score related to the probability of occurrence of a hazard for the hazard assessment.

Probability	Score	Description
Remote	1	Probability of less than 10%
Unlikely	2	Probability between 10% and 35%
Possible	3	Probability between 36% to 64%
Likely	4	Probability between 65% to 90%
Certain	5	Probability above 90%

**Table 2:** Definition of score related to the impact on the human health of hazards for the hazard assessment.

Impact	Score	Description
Insignificant	1	Compounds are categorized as not hazardous or even though hazardous compounds might be present in consumed products, adverse reactions are very unlikely
		Substances that have a positive effect on health are present in the consumed products
Minor	2	Substances are contaminants, but their concentration is regulated. Therefore, no adverse reaction is expected if the regulations are followed. However, they present a hazard and should be monitored
		Substances might have adverse effects when ingested by humans, but there is no regulation or definitive information in this regard
		Substances that have a positive effect on health are present but information about solely their consumption in regards to related animal-based products is lacking
Moderate	3	Substances are contaminants, regulations exist, and they might be present in consumed products
		Substances are contaminants and might be present in consumed products, but no regulations for plant-based meat analogues exist. Until their concentration is also regulated in the investigated products, they possess a moderate threat
Major	4	Significant possibility of adverse reactions, no regulations exist, and substances might be present in the consumed products.
		Susbtance considered a health concern but no epidemiological evidence exists. No regulations exist and substance was found in the consumed products
		Substances that have a positive effect on health are only present in certain products at lower concentrations than animal-based products or other products with excellent nutritional properties
Critical	5	Significant possibility of adverse reactions, no regulations exist, and the substance was found in several related products
		Substances that are known to have a positive effect on health or have excellent nutritional properties are missing

**Table 3:** Definition of code implemented to identify the different hazards in plant-based meat analogues.

Code	Identified Hazard
Chemical hazards	
H1	Heavy metals
H2	Compounds related to protein
H3	Compounds related to protein extraction—Chloropropanol
H4	Compounds related to processing—NNAs and HAAs
H5	Compounds related to processing—AAs
H6	Compounds related to processing—PAHs
H7	Compounds related to processing—Lysinolanine and lanthionine
H8	Binders and gums—Methylcellulose
H9	Binders and gums—Carrageenan
H10	Flavorings and colorants—Leghemoglobin
Allergens	
H11	Allergens
Microbial hazards	
H12	Spore-and non-spore-forming bacteria
H13	Spoilage organisms
H14	Mycotoxins
Nutritional hazards	
H15	Nutritional values
H16	Protein metabolites
H17	Anti-nutritional factors
H18	Isoflavones

**Table 4:** Protein quality calculated using different methods for soybeans and peas as raw materials and their extracted proteins as concentrates (soy protein concentrate (SPC) and pea protein concentrate (PPC)) and isolates (soy protein isolate (SPI) and pea protein isolate (PPI)). The effect of extrusion processing further processing and final formulation on the protein quality is shown for some selected products. Data extracted from references [39,45,64,70,71,72,73,74,75,76].

Parameter	Cooked Soy	Cooked Pea	SPC or SPI	PPC or PPI	Extruded Soy	Plant-Based Beef/Pork	Plant-Based Burger
PDCAAS	73	50–89	86–100	71–93	>54	–	–
DIAAS	65	46–89	84–95	62–70	–	–	71–107
IVPD	49	–	96	54–97	73–96	50–70	–

## Data Availability

No new data were created in this study. Data sharing is not applicable to this article.
